# The breast feeding mother and xenon anaesthesia: four case reports. Breast feeding and xenon anaesthesia

**DOI:** 10.1186/1471-2253-10-1

**Published:** 2010-02-19

**Authors:** Ralph Stuttmann, Claudia Schäfer, Peter Hilbert, Markus R Meyer, Hans H Maurer

**Affiliations:** 1 Department of Anaesthesiology/Intensive and Emergency Medicine/Pain Therapy, BG-Kliniken Bergmannstrost, Merseburger Strasse 165, Halle/Saale, (06112), Germany; 2 Department of Experimental and Clinical Toxicology, Institute of Experimental and Clinical Pharmacology and Toxicology, Saarland University, Kirrberger Str 1, Homburg/Saar, (66421), Germany

## Abstract

**Background:**

Four nursing mothers consented to anaesthesia for urgent surgery only on condition that their ability to breast feed would not be impaired.

**Methods:**

Following induction of general anaesthesia with propofol and remifentanil, 65-69% xenon supplemented with remifentanil was used as an inhalational anaesthetic for maintenance.

**Results:**

After finishing surgery the women could be extubated between 2:52 and 7:22 minutes. The women were fully alert just minutes after extubation and spent about 45 minutes in the recovery room before discharge to a regular ward. They resumed regular breast feeding some time later. The propofol concentration in the blood was measured after 0, 30, 90, and 300 minutes and in the milk after 90 and 300 minutes. Just 90 minutes after extubation, the concentration of propofol in the milk was limited (> 3 mg/l) so that pharmacological effects on the babies were excluded after oral intake. Also, no traces of xenon gas were found in the maternal milk at any time. After propofol induction and maintenance of anaesthesia with xenon in combination with a water-soluble short-acting drug like remifentanil, the concentration of propofol in maternal milk is low (> 3 mg/l 90 min after anesthesia) and harmless after oral intake.

**Conclusions:**

These results, as well as the rapid elimination and absence of metabolism of xenon, are of great interest to nursing mothers. General anaesthesia with propofol for induction only, combined with remifentanil and xenon for maintenance, has not yet been described in breast feeding mothers.

## Background

Surgery and general anaesthesia during pregnancy and lactation are limited in terms of the techniques and drugs that can safely be used. Traces of anaesthetics have little or no effect on older infants, but may cause problems in neonates or pre-term babies predisposed to apnoea [1]. In such cases, breast feeding must be suspended for 12-24 hours. Many mothers attach great importance to exclusive breast feeding with no supplementary bottle feed. In urgent surgery, when the operation cannot be properly scheduled and only general anaesthesia is possible or acceptable to breast feeding mothers, a technique of anaesthesia is desirable which allows the babies to be safely breast fed shortly after surgery. A general recommendation is an interruption of breast feeding for 24 h following anaesthesia but without clear evidence to the author's knowledge. This is disagreeable to mother and baby. Once xenon has been granted marketing approval in 14 countries in Europe, it may become an interesting and new technique for general anaesthesia with a special indication for mothers breast feeding their babies. The aim was to prove this indication in four case reports with control of the blood and milk concentrations of anaesthetic agents.

## Methods

We enrolled four mothers of ASA classification I who attached great importance to nursing and refused regional anaesthesia in urgent surgery. We therefore suggested inhalational anaesthesia with xenon. Each patient consented to this approach, and cases three and four also agreed to blood and milk samples being taken. Written informed consent was obtained one day before anesthesia. The investigation was approved by the institutional research board and the ethic committee of the hospital. The investigation was performed in compliance with German regulatory requirements as well as the internationally valid applicable guidelines.

### Case #1

A 31-year-old mother had suffered a recurrent slipped disc. She was exclusively breast feeding her 5-month-old infant until just before surgery.

### Case #2

A 30-year-old tertipara had suffered a flake fracture of the talus requiring urgent surgery. She was still mostly breast feeding her 5-month-old infant, as well as giving some additional food. The infant was last nursed about 2.5 h before induction of anaesthesia.

### Case #3

A 34-year-old secundipara had developed a ganglion on the hand during pregnancy and was scheduled for surgery after delivery. The infant, who was mainly breast fed, was last nursed by the patient just before attending the hospital for ambulatory surgery.

### Case #4

A 30-year-old mother suffered a fractured fibula requiring urgent osteosynthesis. Her 3-month-old baby was exclusively breast fed, the last time shortly prior to surgery.

The direct perioperative anaesthesiological approach was identical for the four women. After an infusion of balanced Ringer's acetate solution, routine monitoring devices were set up. A Narcotrend^® ^monitor (MT MonitorTechnik GmbH und Co. KG, 24576 Bad Bramstedt, Maienbass 27, Germany) validated for xenon anaesthesia by the author was used to monitor the anaesthetic depth by single-lead EEG [2]. Anaesthesia was induced and controlled by the target controlled infusion technique (TCI) using the Base Primea^® ^unit (Fresenius Kabi GmbH, Bad Homburg, Germany). Propofol was used for induction of anaesthesia based on the dosing model of Schnider [3] and remifentanil was used for analgesia based on the dosing model of Minto [4]. The anaesthesia ventilator was the Dräger Physioflex closed system. Induction of anaesthesia ensued with a calculated effect concentration of 6.5 μg.ml^-1 ^for propofol and 4.5 ng.ml^-1 ^for remifentanil according to the model of Schnider and Minto [3,4]. Following preoxygenation and ventilation with oxygen, Esmeron^® ^(rocuronium bromide) 0.5 mg/kg was injected for neuromuscular blocking to facilitate intubation. Monitoring of the neuromuscular transmission is routine clinical practice. Thereafter, the Physioflex was used for controlled ventilation with an equal setting (tidal volume 8 ml per kg bodyweight and frequency 9 breaths per minute) on the ventilator in all cases. The breathing system was repeatedly flushed with oxygen. Once the endexpiratory oxygen concentration reached about 99% some minutes later, the inspiratory oxygen concentration was reduced to 24%, and xenon insufflation was started by repeated flushing. Propofol infusion was stopped as xenon insufflation was started. The target xenon concentration was between 69% and 65%, and the depth of anaesthesia ranged from sleep stage E0 to E1 on the Narcotrend^® ^witch means sufficient depth of anesthesia [5].

In two patients samples of blood (0, 30, 90, and 300 minutes after anaesthesia) and milk (90 and 300 minutes after anaesthesia) were taken to measure the propofol and xenon concentration. EDTA blood samples were taken, centrifuged, and the plasma stored at - 54°C after shock freezing.

### Sample Preparation and Quantification

After addition of 50 mL butyl acetate, plasma samples (200 mL) and maternal milk samples were extracted for 2 min on a rotary shaker. After centrifugal phase separation (1 min, 10,000 3 g, ambient temperature), the upper layer (organic) was transferred to autosampler vials, and 2 mL injected into the gas chromatography (GC)-mass spectrometry (MS) system.

The samples were analysed using a Hewlett Packard (Agilent, Waldbronn, Germany) HP 6890 Series GC system combined with an HP 5973 series mass selective detector, an HP 6890 Series injector, and an HP Chem Station G1701AA version A.03.00. The GC conditions were as follows: splitless injection mode; column, HP-1 capillary (Agilent), 12 m × 0.2 mm internal diameter, cross-linked methyl silicone, 330 nm film thickness; injection port temperature, 280°C; carrier gas, helium; flow rate 0.6 mL/min; column temperature, initially 100°C for 2 min, increased to 310°C at 40°/min, 310°C for 2.5 min. The MS conditions were as follows: transfer line heater, 280°C; ion source temperature, 230°C; electron mode; ionisation energy, 70 eV; selected ion monitoring (SIM) with the following programme: solvent delay, 3.00 minutes; m/z 117, 163 (target ion), and 178 for propofol. The electron multiplier voltage offset was set to 50 V from 3.00 to 6.00 min.

Full calibration quantification of propofol was carried out by comparison of its peak area in the sample with that of calibration curves, in which the peak areas of spiked calibrators had been plotted against their concentrations (0.1, 0.5, 1.0, 2.5, 5.0, 7.5, 10.0 mg/L).

The maternal milk samples for measuring xenon concentration were injected directly into special glass tubes for gas chromatography and stored at 4°C until the time of estimation.

The analytical measurement of xenon concentration ensued with the help of a commercial ICP-MS (inductively coupled plasma mass spectrometer) in a time resolved measurement mode on several xenon isotope masses. The sample introduction method for xenon measurement was developed by Carsten Pilger (Air Liquide medical GmbH).

## Results

The four operations were uneventful. Table 1 gives information about patient data, time of operation, time of extubation, xenon supply and xenon consumption. Upon completion of all measures, the remifentanil dose was reduced to an effective dose of 1.5 ng.ml^-1^, and once the calculated plasma concentration had decreased to below 1.5 ng.ml^-1^, the ventilatory system was flushed with oxygen, thus simultaneously stopping the supply of xenon. Once the xenon concentration was less than about 20% and all patients opened their eyes and could follow commands, their tracheas were extubated. The patients were then transferred to the recovery room and monitored for about 45 minutes before being returned to the ward fully alert with permission to eat and drink. None of the women experienced nausea or vomiting at any time.

**Table 1:** Details of patient age, OP time, xenon duration, extubation time and xenon consumption.

Patient	Age [years]	Weight (kg)	OP time [min]	Xenon time [min]	Extubation time [min]	Xenon consumption [l]
1	31	78	40	60	2:52	15.00
2	30	64	45	70	7:22	15.33
3	33	60	35	57	6:30	10.00
4	30	58	35	59	4:51	8.28

In case #1, the mother resumed nursing at 12:00 noon (2 hours and 50 min after anesthesia). In case #2, the infant was bottle fed at midday, and regular nursing was resumed in the evening (4 hours and 40 min after anesthesia). The third baby was breast fed 5 hours after the end of anaesthesia, and the fourth baby after 90 minutes. During the first nursing session all babies were observed very carefully for dizziness and drowsiness, but no such signs were seen. The subsequent period at home was completely uneventful, both for the babies and their mothers.

Table 2 gives information about the concentration of propofol in the blood and breast milk as well as xenon in the breast milk. The blood concentration of propofol is low just 30 minutes after extubation. After 90 and 300 minutes, propofol was measured in the milk and calculated for a volume of 150 ml (Table 3). No traces of xenon could be detected.

**Table 2:** Concentration of propofol and xenon in the blood and maternal milk after 0, 30, 90 and 300 minutes post extubation

			Time (t) after extubation [min]			
	Sample	Case	0	30	90	300
Propofol [mg/l]	Blood	#3	0.47	0.47	0.21	0.12
		#4	0.48	0.45	0.16	0.11
	Maternal milk	#3				0.13
		#4			2.78	0.84
Xenon	Maternal milk	#3	0			0
		#4	0			0

**Table 3:** Initial doses of propofol [mg] and related concentrations [mg], as well as percentages in the maternal milk after 90 and 300 minutes related to 150 ml

Case	∑ Propofol IV [mg]	mg/150 ml maternal milk	
		90 min	300 min
#3	350		0.0195 (0.006%)
#4	443	0.417 (0.09%)	0.126 (0.028%)

## Discussion

Xenon was discovered in 1898 by Ramsay and Travers. It is the only noble gas with anaesthetic properties under normobaric conditions [6]. Cullen successfully performed general anaesthesia with xenon for the first time in 1951[7]. Xenon comes very close to being the ideal anaesthetic [8-12]. Its unique properties include a rapid onset and offset of action [13] and a recovery index with good arousal quality [14].

Breast feeding is known to have significant benefits in terms of infant development. The World Health Organisation (WHO) recommends exclusive breast feeding for 6 months and partial continuation into the second year of life [15]. How safe is breast milk for the infant, however, if the mother has taken medication? Unfortunately, recommendations tend to be based on case reports, theoretical risks or case studies [16].

The breast milk accumulation in 24 hours of most drugs is rarely greater than 1-2% of the original dose [17]. Drugs with high lipid solubility like propofol are found at higher concentrations in breast milk than in plasma dependent on the time and amount of milk production during propofol administration. This could be confirmed when determining the levels of propofol in the blood and maternal milk 90 and 300 minutes post extubation. Water-soluble drugs with low molecular weight (< 200) are excreted in the breast milk unimpeded, resulting in identical milk and plasma concentrations. As a result of the difference between plasma pH (7.4) and milk pH (6.5-7.09), weak acids are excreted in smaller quantities in milk, whilst weak bases are excreted in higher concentrations. High protein binding as is the case with remifentanil (70%) is associated with low milk concentrations, since only the free fraction of a drug can enter the breast milk. Remifentanil is metabolised within minutes by nonspecific plasma esterases and theoretically excretion in the mother's milk is very unlikely. A high degree of ionisation as with Esmeron results in an exceedingly low drug concentration in the milk [17-19].

Xenon, a noble gas, is absorbed or exhaled via the lungs. It does not form any compounds in the body and is not metabolised. The partition coefficients increase from xenon to nitrous oxide, sevoflurane and isoflurane. The blood/gas partition coefficient is the main determinant of the rate of onset and offset of action of an inhalational anaesthetic. The lower the blood/gas partition coefficient, the faster these processes. The oil/gas partition coefficient or the lipid solubility of an inhalational anaesthetic is a measure of its potency: a high lipid solubility means high potency but also a longer emergence time [6,20]. The low lipid solubility of xenon explains its high minimal alveolar concentration (MAC) as well as its short recovery times.

Xenon has excellent physicochemical properties, explaining its anaesthetic effect under normobaric conditions. The MAC of xenon ranges from 51% to 69% [21-23]. The oil/water partition coefficient of xenon is around 20. Based on the physical properties of xenon and a breast-milk fat concentration of approximately 51%, dissolution of xenon in human milk cannot be ruled out completely, but would theoretically be expected to be lower than that of sevoflurane or isoflurane, respectively. Since an accumulation process in breast milk is not possible based on diffusion gradients and because the residual concentration of xenon is exhaled from the mother's body in a matter of minutes, considerations suggest that xenon is no longer detectable in the milk a few minutes after emergence. This suggestion is consistent with the concentration of xenon we measured in the breast milk (see Table 2). Furthermore it can be assumed that during the suction process traces of xenon diffuse from the milk into the air within seconds, so that the question of oral bioavailability does not arise.

The unique benefits of inhalational anaesthesia with xenon in nursing mothers include rapid elimination in a matter of minutes, the absence of metabolism of this noble gas and analgetic properties.

Accounts in the literature on the anaesthetic approach to nursing mothers are sparse [19,24,25]; guidelines do not reflect this setting, and textbooks recommend interruption of breast feeding for 24 h. Nor do the propofol and remifentanil SPCs (Summary of Product Characteristics) comment on usage during lactation.

Remifentanil, co-administered with xenon to ensure analgesia, is rapidly metabolised postoperatively and has a plasma half-life of 3-10 minutes. At a calculated effect concentration of less than 1.5 ng/ml read from the Base Primea controller at extubation, it is therefore safe to assume that this opioid is no longer active. No data is available on the excretion of remifentanil in breast milk (information provided by the manufacturer).

Propofol was used only for induction. Nitsun and colleagues found that the breast-milk drug concentration was 0.015% of the original dose over a 24 h measuring period [26]. This represents the upper limit based on the assumption that the infant ingests the total milk volume produced during a 24 h period and that propofol has 100% oral bioavailability. In light of the two cases in which propofol was measured in the breast milk, this would imply that a maximum of 52.5 μg/24 h could be absorbed in case 3 and 66.45 μg/24 h in case 4. Assuming that a baby drinks about 150 ml per breast feed, this amount - when converted in line with the concentrations in the maternal milk after 90 and 300 minutes - is equivalent to the doses given in Table 3. In case 4 we could detect a clear deviation from the claim made by Nitsun et al after just 90 and 300 minutes, with 0.09% and 0.028% of the measured concentration in the breast milk related to the initial dose. Hence the findings published by Nitsun et al do not apply to our patients and require further investigation in a larger population. Nevertheless, it can be presumed that orally, the baby ingests an extremely low concentration of propofol. Taken also into account the very low oral bioavailability babies will be exposed to a negligible quantity of propofol during breast feeding.

## Conclusion

Xenon anaesthesia is an alternative method for nursing mothers, enabling breast feeding to be resumed immediately after surgery without harming the baby. This is also the case in surgery lasting several hours, since propofol is given only for induction. When given inhalationally for anaesthetic maintenance, xenon does not accumulate and is exhaled within a few minutes irrespective of the duration of inhalation. The measurements clearly demonstrate that, 90 minutes after extubation, neither amounts of lipid-soluble propofol nor xenon can be detected in milk. The unique benefits of inhalational anaesthesia with xenon in nursing mothers include rapid elimination in a matter of minutes, the absence of metabolism of this noble gas and analgetic properties. Xenon anaesthesia combined with short-acting opioids may be a new, alternative anaesthetic technique recommendable for breast feeding mothers on paediatric grounds.

## Competing interests

The authors declare that they have no competing interests.

## Authors' contributions

RS designed the investigation, reviewed the literature and drafted the manuscript. CS and PH performed the anaesthesia, helped to draft the manuscript and took care of the patients. MRM and HHM performed the lab-tests. All authors read and approved the manuscript.

## Pre-publication history

The pre-publication history for this paper can be accessed here:

http://www.biomedcentral.com/1471-2253/10/1/prepub
